# From sound waves to molecular and cellular mechanisms: Understanding noise‑induced hearing loss and pioneering preventive approaches (Review)

**DOI:** 10.3892/mi.2024.184

**Published:** 2024-07-30

**Authors:** Pinelopi Samara, Michail Athanasopoulos, Nikolaos Markatos, Ioannis Athanasopoulos

**Affiliations:** 1 Children's Oncology Unit ‘Marianna V. Vardinoyannis-ELPIDA’, Aghia Sophia Children's Hospital, 11527 Athens, Greece; 2 Otolaryngology-Head and Neck Surgery, Athens Pediatric Center, 15125 Athens, Greece

**Keywords:** noise-induced hearing loss, hair cells, cochlea, animal models, preventive measures

## Abstract

Noise-induced hearing loss (NIHL) is a significant and urgent global public health concern, arising from prolonged exposure to elevated levels of noise. This auditory impairment harms delicate inner ear structures, particularly the essential hair cells transmitting auditory signals to the brain. Recognized by the World Health Organization as a major contributor to worldwide hearing loss, NIHL requires a comprehensive examination of its molecular and cellular mechanisms. Animal models emerge as indispensable tools for unraveling these intricacies, allowing researchers to simulate and study the impact of noise exposure on auditory structures, shedding light on the interplay of oxidative stress, inflammation and immune responses-crucial factors in NIHL progression. The present review focuses on elucidating the molecular mechanisms of NIHL, with a specific emphasis on findings derived from animal models, alongside the exploration of thorough preventive strategies, including protective measures and probing potential interventions. Understanding the molecular underpinnings not only provides insight into targeted treatment approaches, but also unlocks pathways for exploring and implementing preventive actions. This approach not only deepens the current comprehension of NIHL, but also has the potential to influence the shaping of public health policies, offering a nuanced perspective on this prevalent auditory disorder.

## 1. Introduction

Noise-induced hearing loss (NIHL) has become a growing global public health concern, drawing attention due to its widespread prevalence and significant impact on individuals worldwide (1). The adverse effects of prolonged exposure to excessive noise levels on auditory function have led to a substantial increase in research interests within the scientific community. This type of hearing impairment arises from continuous exposure to elevated noise levels commonly found in various occupational and recreational settings. While individual sensitivity varies, exposure to sound intensities exceeding 85 dB can result in NIHL (2).

NIHL affects various critical components within the inner ear. Extended exposure to loud sounds stresses key elements, such as hair cells and supporting cells, each playing a vital role in auditory function. The damage extends to structures, such as the stria vascularis, crucial for maintaining the potential of the inner ear, and the tectorial membrane involved in mechanical stimulation. Notably, the inner hair cell synapse is vulnerable from an early stage, preceding observable hair cell loss. Additionally, spiral ganglion cells, responsible for transmitting auditory signals, may also be affected (3). The World Health Organization recognizes NIHL as a leading contributor to global hearing loss (4), highlighting the urgent need for a comprehensive understanding of its molecular and cellular mechanisms.

NIHL manifests as progressive hearing loss, and temporary threshold shifts are possible following exposure to transient or moderate noise. Prolonged exposure to harmful noise can result in permanent hearing loss, predominantly affecting high frequency hearing. Acute acoustic trauma from extremely high noise levels, such as a cannon firing can cause immediate severe damage, leading to sudden deafness, tinnitus and potential ear injuries (5). The condition markedly affects the daily lives of patients, contributing to chronic symptoms, such as tinnitus, headaches, dizziness and insomnia, often exacerbating feelings of depression and isolation.

The present review aimed to bridge the gap between the macroscopic perspective of NIHL as a burgeoning public health challenge, and the microscopic exploration of the intricate molecular and cellular processes that underlie its onset and progression. To compile the relevant literature, searches were conducted across multiple electronic databases, including PubMed, Scopus, Web of Science and Google Scholar. A combination of keywords and phrases were utilized, such as ‘noise-induced hearing loss’, ‘molecular mechanisms of hearing loss’, ‘preventive strategies for hearing loss’ and ‘animal models of hearing loss’. The selection criteria focused on peer-reviewed articles published in the English language within the past 25 years. The objective of the present review encompasses a dual aim: i) To gain a profound understanding of the intricacies of NIHL at the molecular level; and ii) to contribute to the advance of targeted interventions and preventive strategies that offer a nuanced perspective on this pervasive auditory disorder. This approach intends to pave the way for therapeutic advancements, alleviating the burden of NIHL on individuals and society at large. The uncovering of the molecular and cellular mechanisms of NIHL is promising for the shaping of future public health policies and interventions to mitigate the widespread impact of noise-induced auditory impairment.

## 2. Evolution and milestones in NIHL research: Technological breakthroughs in understanding its biological basis

The study of NIHL has its roots in the industrial revolution when the surge in mechanized activities exposed workers to unprecedented levels of occupational noise. Early observations of hearing impairment among industrial workers sparked an interest in understanding the association between noise exposure and auditory function (6). Over the years, NIHL research has evolved in a sophisticated scientific discipline. The development of NIHL research can be delineated into distinct phases, reflecting advancements in scientific methodologies and increasing awareness of the prevalence of this condition. Initially, research primarily focused on clinical manifestations, gradually shifting towards more comprehensive investigations into the physiological and molecular underpinnings of NIHL. The advent of advanced diagnostic tools and imaging technologies has facilitated a deeper understanding of the intricate mechanisms involved in noise-induced damage to the auditory system.

Throughout the evolution of NIHL research, several key milestones and breakthroughs have markedly shaped the understanding of the biological basis of this auditory disorder. Fundamental discoveries include the identification of specific cellular structures, such as the vulnerable hair cells within the inner ear as primary targets of noise-induced damage (7). Molecular investigations have elucidated the role of oxidative stress, inflammation and genetic predispositions in amplifying susceptibility to NIHL (8). These milestones represent crucial steppingstones toward unraveling the complexity of NIHL at the cellular and molecular level. Technological advancements have played a pivotal role in advancing NIHL research, with the use of animal models standing out as a noteworthy strategy. Cutting-edge technologies, such as genome editing and functional imaging (9) have empowered scientists with the methods to delve deeper into the intricate cellular processes and molecular pathways implicated in noise-induced auditory damage. The integration of these technological tools with traditional research approaches has enhanced the translatability of findings from bench to bedside, fostering the development of targeted interventions and preventive strategies.

## 3. Animal models in NIHL research: Strengths and limitations of different animal models

Animal models offer a controlled environment for investigating the progression of NIHL. Researchers can manipulate variables, observe outcomes with precision, and use these models as essential tools to identify therapeutic targets and evaluate the effects of new drugs. Moreover, a number of molecular and cellular pathways related to hearing are conserved across species (10). Therefore, findings in animals can provide valuable insight into human auditory function and pathology. Commonly used species include rodents, such as rats, mice, guinea pigs (11) and chinchillas (12), as well as fish, notably zebrafish, and certain non-human primates, such as macaques, marmosets and monkeys (10).

Rodents are commonly used in hearing research due to their cost-effectiveness, widespread availability and the feasibility of conducting large-scale experiments. Rodents exhibit auditory systems closely resembling those of humans, particularly in the structure and function of the cochlea. They are well-suited for a range of electrophysiological hearing tests and behavioral assessments tailored to evaluate auditory function. Rat hearing matures postnatally, presenting research opportunities, such as studying NIHL compared with the prenatally matured cochlea in humans (13). Genetically modifiable mice have gained prominence, offering valuable genetic and molecular insight into factors such as sex, circadian cycle, coat color, age and genetic background that influence NIHL (14). The mouse cochlea has a similar number of turns to the human cochlea; however, its cochlear duct is notably shorter. While mice replicate human cochlear anatomy, physiology and pathology, differences in hearing range, including the fact that mice typically have a higher hearing frequency range than humans, and susceptibility to noise-induced hearing cell loss need to be considered when translating results to clinical practice (15). Mice play a crucial role in validating susceptibility genes identified in human NIHL and in discovering new susceptibility and resistance genes. Ongoing genetic mapping studies aim to identify potential DNA regions conferring noise resistance in inbred mouse strains (16).

Zebrafish lack outer and middle ears, but possess inner ears with hair cells. They also rely on a lateral line system with neuromasts that also contain hair cells sensitive to water movements. Despite the absence of a mammalian cochlea, this distinctive sensory system enables them to detect vibrations and navigate their aquatic environment effectively (17). Additionally, owing to the transparency of embryos and larvae, the real time observation of inner ear development and function in zebrafish provides a respected method for visualizing the impact of noise on delicate inner ear structures. Notably, zebrafish exhibit regenerative capabilities in their inner ear hair cells, offering insight into the potential for hair cell regeneration after noise-induced damage, a process limited in mammals (18).

Non-human primates, such as macaques, exhibit marked physiological and anatomical parallels with humans, particularly in the auditory system. This resemblance positions them as invaluable assets in research, serving as a vital link between rodent studies and potential applications in humans. Their advanced cognitive capacities and behavioral similarities enhance investigations into complex auditory processing, offering a more comprehensive understanding of how auditory stimuli are perceived and processed (19).

It is noteworthy that the choice of model organism depends on the specific research questions and goals (Table I). The use of multiple species in complementary studies helps researchers gain a broad understanding of the mechanisms underlying NIHL and develop potential therapeutic interventions that may translate to clinical applications in humans.

## 4. Molecular pathways in NIHL

### Oxidative stress, inflammation and immune responses in the progression of NIHL

**Figure 1:**
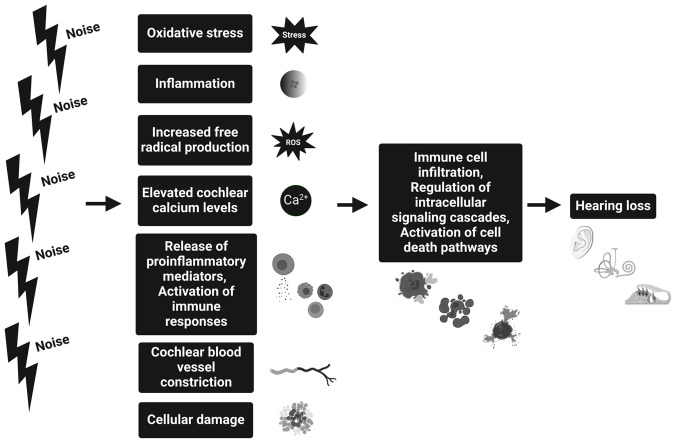
Pathophysiology of noise-induced hearing loss. Prolonged exposure to loud noise triggers inflammation and oxidative stress, initiating inflammatory events in the cochlea. Elevated cochlear calcium and free radicals, induced by noise, further activate immune processes, leading to direct and calcium-mediated vasoconstriction. This process causes cellular damage and activation of cell death pathways, resulting in hearing impairment. The figure was created using BioRender.com.

The progression of NIHL involves an intricate interplay of biological processes, among which oxidative stress, inflammation and immune responses play pivotal roles (Fig. 1). Excessive noise triggers vasoconstriction in cochlear blood vessels, reducing inner ear blood flow. This primes the cochlea for reactive oxygen species (ROS) production during reperfusion after noise exposure ceases. ROS overproduction during reperfusion, fueled by the sudden influx of oxygen-rich blood, is facilitated by inflammatory activation and mitochondrial disruption. This oxidative stress leads to cellular damage, particularly affecting hair cells due to their prolonged exposure within the inner ear (20). Elevated free Ca^2+^ in cochlear hair cells, resulting from extracellular entry and subsequent release from intracellular stores, contributes to cytoplasmic ROS accumulation, the potential activation of cell death pathways and the modulation of intracellular signaling cascades, emphasizing its involvement in noise-induced damage to hair cells (21). These free radicals, resulting from an imbalance between ROS production and antioxidant defense mechanisms, have the potential to harm biological membranes, DNA and induce apoptosis. Following exposure to noise, the cochlea responds by upregulating various antioxidant enzymes to counterbalance the production of free radicals. Glutathione, while not classified as an enzyme, serves as a pivotal antioxidant molecule, playing a crucial role in neutralizing free radicals and safeguarding cells against oxidative damage (22). Increased transcription and translation of key antioxidants such as superoxide dismutase (SOD) type 1 and heme oxygenase-1 genes have been observed in mice (23,24). Tuerdi *et al* (25) revealed the crucial role of Mn-SOD, a mitochondrial antioxidant enzyme, in protecting the cochlea from NIHL. Using Mn-SOD knockout mice, their study found worse hearing thresholds and greater outer hair cell damage after exposure to noise at 120 dB. These findings underscore the vital role of Mn-SOD in mitigating the impact of ROS and preserving auditory function in response to noise.

Concomitantly, inflammation emerges as a significant contributor to the progression of NIHL. The inner ear exhibits robust immune capabilities, with non-immune cells producing immunoregulatory molecules and various immune cells participating in the immune and inflammatory response (26). Post-noise exposure, immune and inflammatory reactions in the cochlea manifest within 1-2 days, with peak at 3-7 days, and gradually subside. The most substantial hearing loss occurs within a few hours, suggesting that while peak immune and inflammatory reactions happen at 3-7 days, hearing loss cannot be solely attributed to these responses. These patterns are consistently observed in diverse mouse models, including wild type and transgenic mice across various strains, as highlighted in a review by Wood and Zuo (27). The release of pro-inflammatory mediators in response to noise-induced trauma amplifies tissue damage and exacerbates the degeneration of auditory structures. This complex cascade involves immune cell infiltration and activation of signaling pathways, collectively contributing to the detrimental effects on auditory function. Additionally, this process results in the production of pro-inflammatory cytokines and chemokines such as tumor necrosis factor-α (TNF-α), interleukin-1β and chemokine ligand 2 in the cochlea (28). The recruitment of macrophages into the cochlea intensifies the potential for noise damage through inflammation. Notably, there are marked disparities in macrophage responses between acute and chronic cochlear noise-induced pathogenesis (29). Acute noise overexposure triggers rapid monocyte infiltration, making infiltrated macrophages the primary executors of inner ear immune activities. By contrast, chronic lower-level noise stress predominantly engages mature resident tissue macrophages in the cochlear immune response. Understanding and modulating these oxidative stress, inflammatory and immune responses represent promising avenues for developing interventions to ease the impact of NIHL.

### Genetic susceptibility to NIHL

The susceptibility to NIHL varies among individuals, and genetic factors play a notable role in this diversity. This refers to the inherent tendency of an individual to be more prone to NIHL based on their genetic characteristics. Identifying genes related to noise susceptibility in humans is challenging due to the difficulty in gathering subjects exposed to identical noise conditions. Consequently, researchers have turned to screening for single nucleotide polymorphisms. While the majority of mutations do not affect normal gene function, point mutations in the gene or its regulatory sequence can affect its functionality. Multiple genes associated with auditory function, oxidative stress response, and cellular repair mechanisms have been implicated in influencing an individual's predisposition to NIHL. Notable genes include glutathione S-transferase Mu 1 and glutathione S-transferase θ1 involved in detoxification processes, potassium voltage-gated channel subfamily q member 4 associated with ion balance regulation, transmembrane channel-like protein isoform 1 essential for cilia structure, heat shock protein (HSP) 70 critical for stress response, X-ray repair cross complementing 1 protein responsible for DNA repair and caspase-3 related to apoptosis. These genes play crucial roles in orchestrating the intricate balance of cochlear resilience and susceptibility to noise-induced damage (30). Genetic polymorphisms affecting the expression or function of these genes can either enhance resilience or elevate susceptibility to noise-induced damage.

Exploration of the genetic landscape in NIHL has uncovered potential biomarkers and therapeutic targets. Genetic variations influencing the metabolism of ROS, regulation of inflammatory responses and maintenance of structural integrity in the inner ear play a crucial role in determining susceptibility to NIHL. While a wide spectrum of >200 genes is associated with deafness, the subset relevant to NIHL focuses on genes associated with oxidative stress, potassium metabolism, specific protein encoding and the family of HSP genes (31). As the understanding of the genetic determinants of NIHL advances, personalized approaches to prevention and treatment may emerge in the future. These approaches could customize interventions according to the genetic profile of an individual, enhancing effectiveness and outcomes. This genetic perspective may devise strategies to identify individuals at a higher risk of NIHL, facilitating early intervention.

## 5. Cellular responses to noise exposure

### Cellular changes in response to noise trauma involve hair cells, supporting cells and neurons, contributing to the pathogenesis of NIHL

NIHL initiates a cascade of cellular transformations in the auditory system, resulting in permanent damage and hearing impairment. The transmission of intense noise energy to the inner ear triggers pronounced fluctuations in perilymph and endolymph, exerting formidable forces on the basilar and tectorial membrane. Specialized sensory cells, hair cells, crucial for transducing sound vibrations into electrical signals, play a pivotal role in this process. Prolonged or intense exposure to loud noises typically >85 dB can cause mechanical damage, leading to the separation of cilia on hair cells and hindering effective reception of vibrational stimuli. This trauma extends beyond hair cells, impacting supporting cells essential for cochlear architecture and function, thereby jeopardizing the structural integrity of the auditory system. The disruption of supporting cells not only unsettles the immediate cellular environment, but also intensifies the damage to hair cells. The oscillation of the lymph can detach hair cells from the basilar membrane, disturbing ribbon synapses, compromising the encoding ability of hair cells and manifesting as difficulties in language comprehension in noisy environments (32,33).

Continued exposure to noise, at a level of 85 dB for a duration >8 h can result in the destruction of both inner and outer hair cells responsible for perceiving vibrations and amplifying sound, respectively (34). Considering the non-regenerative nature of hair cells, preventing intense noise exposure emerges as a crucial measure. Understanding the mechanistic basis of NIHL emphasizes the importance of addressing factors contributing to cochlear damage, particularly in safeguarding the irreplaceable nature of hair cells.

### Noise exposure activates cell death pathways

The cumulative effects of mechanical damage, oxidative stress and inflammation can induce cell death in the auditory system (21). Apoptosis, a regulated process aimed at eliminating damaged cells, is activated in response to noise exposure, particularly within the cochlea, targeting sensory hair cells and supporting cells. The uncontrolled activation of apoptotic pathways due to intense vibrations of loud sounds contributes to permanent loss of functional cells, impairing hearing. Autophagy, a cellular recycling process, acts protectively by degrading damaged components in response to noise exposure (35). Triggered by clear damaged organelles and maintain cellular integrity, autophagy is a dynamic process. However, excessive or dysregulated autophagy can also lead to cell death. The intricate interplay between apoptosis and autophagy in the auditory system following noise exposure remains a subject of ongoing research, highlighting the complexity of cellular responses to auditory stressors. Additionally, cell loss may result in scar tissue formation, further compromising the normal functioning of auditory structures.

Studies conducted over the past decade suggest that necrosis, the uncontrolled cell death with membrane swelling, and pyroptosis, a process associated with inflammation and membrane pore formation, also contribute to cochlear cell death from noise. Specifically, the study by Zheng *et al* (36) 10 years prior, unveiled potential therapeutic strategies for mitigating NIHL in mice by targeting apoptotic and necrotic pathways in hair cells through pharmacological inhibitors and receptor-interacting protein 3-silencing techniques. Furthermore, a recent study by Sai *et al* (37) in miniature pigs revealed the elevated expression of NLR family pyrin domain containing 3 (NLRP3), interleukin-1β, interleukin-18 and cleaved-caspase-1 in the cochlea after exposure to white noise of 120 dB, indicating the activation of NLRP3 inflammasomes as a significant contributor to NIHL.

## 6. Synaptic mechanisms and neural plasticity in NIHL

### Noise-induced damage affects synaptic connections in the auditory pathway

The detrimental impact of noise extends beyond the outer and middle ear, affecting the intricate synapses between hair cells and auditory nerve fibers. Notably, cochlear synaptopathy, also known as hidden hearing loss, can lead to subtle hearing deficits, particularly in challenging listening situations. Unlike traditional hearing loss, which affects hair cells directly, cochlear synaptopathy primarily disrupts the neural connections between inner hair cells and auditory nerve fibers, impairing the transmission of auditory signals to the brain. Synaptic alterations, as well as loss of the medial olivocochlear reflex, occurring prior to hair cell loss, play a marked role in the cascade of events triggered by noise exposure during the initial stages of NIHL. These alterations encompass reduced synaptic numbers, altered neurotransmitter release and disrupted cell communication, collectively impairing signal transmission (32). This pervasive influence of noise transcends the cochlea, reaching higher-level brain regions.

Noise affects the brainstem and cortex, disrupting the processing of auditory information. In the brainstem, noise exposure induces alterations in neural circuits responsible for relaying auditory signals to higher brain centers, potentially leading to distorted representations of sound in the auditory pathway. In parallel, the brainstem demonstrates plasticity through central compensation in response to noise, with ensuing changes in neural signals potentially causing hyperactivity in the central auditory system and contributing to the perception of tinnitus (38). Similarly, the cortex, a pivotal center for complex auditory processing, experiences disruptions in neural activity and connectivity due to prolonged exposure to intense noise, contributing to difficulties in identifying and interpreting sounds (39).

Notably, NIHL influences the hippocampus, a region traditionally linked to memory and cognitive functions. Despite its location outside the classical auditory pathway, the hippocampus undergoes altered neurogenesis and displays spatial memory deficits in response to acoustic input influenced by noise exposure. Prolonged exposure to intense noise elevates corticosterone levels, impacting neurogenesis, while permanent hearing loss results in transient corticosterone elevation. The hippocampus, responsible for regulating corticosterone release, faces challenges, contributing to cognitive decline associated with NIHL (40). This intricate interplay between noise-induced changes in the auditory system and cognitive functions emphasizes the profound consequences of prolonged exposure to intense noise.

### Potential for neural plasticity following noise exposure

The potential for neural plasticity following noise exposure is a study area of increasing interest. Emerging research suggests that the nervous system, particularly the auditory pathways, possesses the ability to undergo structural and functional changes in response to noise-induced damage. Neural plasticity, the capacity of the brain to reorganize and adapt, plays a crucial role in post-noise exposure adaptation (41). Studies have indicated that the auditory system can exhibit plasticity at various levels, from the cochlea to higher auditory centers in the brain (42-44). These mechanisms encompass synaptic plasticity, changes in neural connectivity and the use of alternative pathways to offset impairments. Additionally, the brain demonstrates central auditory processing reorganization, adapting its strategies to enhance perception. This adaptability markedly improves auditory function, particularly when combined with rehabilitative interventions, such as auditory training or the use of hearing aids.

More specifically, plastic adaptations occur in higher auditory centers, characterized by synaptic remodeling and altered neural connectivity. Neurodegenerative changes in the auditory nerve post-noise exposure trigger neuroplastic alterations within the cochlear nucleus, reshaping synaptic structure and function. Molecular mechanisms, including the upregulation of genes, such as Bdnf, Homer-1 and Grin1, drive this synaptic reorganization. These mechanisms were previously investigated using a rat model, revealing changes in synaptic plasticity genes, with more pronounced alterations observed in the ventral cochlear nucleus. The majority of these changes manifested 28 days post-noise damage (45). While the extent of improvement varies among individuals and depends on factors such as age, the duration of noise exposure and the severity of hearing damage, the potential for neural plasticity underscores the importance of early intervention and rehabilitation strategies. Understanding these processes provides valuable insight which may aid in the development of targeted therapies that harness the inherent ability of the brain to adapt after noise-induced hearing loss.

## 7. Diagnosis of NIHL

### Diagnostic methods for NIHL

The optimal approach for detecting NIHL necessitates a comprehensive audiometric assessment, employing diverse tests to delineate the extent and characteristics of hearing impairment, along with an evaluation of individual exposure history. This multifaceted process confirms audiometric characteristics related to noise trauma, while ruling out other potential pathologies. Pure-tone audiometry remains a cornerstone in diagnosing NIHL, constructing an audiogram that reveals the nuanced pattern of hearing loss across frequencies (8).

NIHL is identifiable by characteristic audiometric patterns, featuring preserved hearing in low-to-mid frequencies, an abrupt decline >3,000 Hz, particularly at 4,000 Hz, and a modest recovery in higher frequencies (46). With prolonged exposure to noise, the decline intensifies, impacting both higher and lower frequencies, and deepening the characteristic notch in the audiogram.

Complementary to this, speech audiometry evaluates the capacity of an individual to understand spoken words at different volume levels. Tympanometry, exploring middle ear function and eardrum mobility, exposes potential abnormalities through alterations in air pressure within the ear canal. Otoacoustic emission testing captures the sounds emitted by the inner ear in response to stimuli, indicating the presence of cochlear hair cell damage. Auditory brainstem response testing examines the electrical activity in response to sound stimuli, uncovering potential anomalies in the auditory nerve and brainstem pathways. High-frequency audiometry, concentrating on regions susceptible to early noise-induced damage, supplements the diagnostic arsenal (47). Self-reported questionnaires can enrich the evaluation by capturing historical data on noise exposure and detailing the pragmatic impact of hearing loss on daily life. This integrative approach, encompassing both clinical assessments and contextual information, enables a comprehensive diagnosis of NIHL within the broader framework of audiological health.

### Pediatric NIHL diagnosis: Early detection and risk assessment

The diagnosis of NIHL in pediatric populations presents distinct challenges due to developmental considerations and the potential for long-term consequences (48). The auditory systems of children are still evolving, necessitating nuanced diagnostic strategies tailored to their unique developmental stages. This includes using specialized hearing tests sensitive to age-related variations in hearing sensitivity and auditory processing (49). Moreover, acquiring accurate information about the exposure of a child to noise is challenging, involving a mix of recreational and environmental factors, further complicating the diagnostic process. The untreated repercussions of NIHL in children extend beyond immediate auditory impairments, profoundly impacting critical aspects of development, including language acquisition, academic performance and overall cognitive abilities. Recognizing the urgency of early identification and intervention is paramount to mitigate these far-reaching consequences. Hence, specialized diagnostic approaches are imperative, integrating multidisciplinary evaluations that consider developmental milestones and individual differences in susceptibility to noise-induced damage. By addressing the specific features of the diagnosis of pediatric NIHL through meticulous assessment techniques and tailored interventions, healthcare professionals can effectively safeguard the long-term auditory health and overall well-being of children.

## 8. Therapeutic approaches and interventions: Current and future pharmacological and surgical strategies

There are currently no widely acknowledged and scientifically validated treatments specifically designed for NIHL contrary to the availability of such treatments for other types of hearing loss. Ongoing research is exploring innovative approaches for both current and future interventions, focusing on anti-inflammatory, antioxidant, anti-excitatory and anti-apoptotic pathways. Promising treatments for NIHL include agents such as D-methionine, vitamin B12, folic acid, coenzyme Q10, N-acetylcysteine (NAC), ebselen and HK-2, selected for their antioxidant or anti-inflammatory properties.

A recent systematic review thoroughly investigated the protective effects of specific vitamins and antioxidants against occupational NIHL, highlighting the significance of such interventions (50). These vitamins and antioxidants have different mechanisms of action that contribute to their protective effects. Vitamin B12 and folic acid play crucial roles in DNA synthesis and repair, as well as in maintaining nerve health and red blood cell formation, which aids in cellular recovery and repair after noise-induced damage (51). NAC functions as an antioxidant by replenishing intracellular glutathione levels, thereby reducing oxidative stress and inflammation (52). Retinoic acid, a metabolite of vitamin A, has shown promise in protecting the cochlea and promoting auditory recovery in noise-exposed mice through mechanisms involving cellular differentiation and regeneration. Vitamin E functions as a lipid-soluble antioxidant, reducing cell death and auditory threshold shifts in both guinea pigs and humans after noise exposure by protecting cell membranes from oxidative damage (53). Similarly, vitamin C, a water-soluble antioxidant, mitigates threshold shifts in animal models by scavenging free radicals and reducing oxidative stress, although its oral administration had limited efficacy, likely due to issues with compliance and bioavailability (54). While vitamin B12, folic acid and NAC have demonstrated notable protective effects, the efficacy of vitamins A, C and E remain inconclusive due to variability in study results and challenges in achieving effective concentrations *in vivo* (50).

In the study by Alvarado *et al* (55), the oral supplementation of vitamins A, C and E combined with Mg^2+^ in rats was shown to reduce auditory threshold shifts and increase the survival of sensory hair cells, possibly by modulating antioxidant enzymes and apoptosis-related proteins in the cochlea, indicating a protective mechanism against NIHL. NAC has shown promise in enhancing glutathione production and reducing auditory threshold shifts in both animal models (56,57) and human studies (58,59). However, determining the precise doses for humans requires the careful consideration of variables, such as age, weight and health status, and the type and severity of noise trauma, in consultation with healthcare professionals to avoid potential risks, including paradoxical increases in oxidative stress (60). Similarly, ebselen, a synthetic compound known for its robust antioxidant properties, mimics the activity of glutathione peroxidase, a crucial enzyme in cellular defense against oxidative stress. By neutralizing harmful free radicals and ROS, ebselen helps maintain cellular integrity and protects against oxidative damage. Notably, in healthy adults, when administered at a dose of 400 mg twice daily, it has demonstrated marked efficacy in reducing temporary threshold shifts at 4 kHz, indicating its promising role in preventing NIHL (61). Additionally, HK-2, a novel member of the synthetic multifunctional redox modulator class, emerges as a promising intervention for oxidative stress-related conditions such as NIHL. Through its potent redox-modulating capabilities, HK-2 effectively preserves cochlear function and maintains hair cell integrity in both *in vitro* and *in vivo* studies. These findings underscore the potential of HK-2 to act as a potent therapeutic measure against NIHL induced by oxidative stress (62).

Studies have also explored the potential of hyperbaric oxygen therapy (HBO2) and corticosteroids, either alone or in combination, in managing acute acoustic trauma, with a focus on alleviating inner ear inflammation. Holy *et al* (63) investigated the effects of early treatment and HBO2 on hearing recovery in military personnel with acute acoustic trauma. Following the analysis of 108 patients between 2004 and 2019, their study found that prompt treatment within 7 days notably boosted the chances of hearing improvement. Corticosteroids and HBO2 were both effective, particularly when administered promptly. Immediate corticosteroid therapy post-acute acoustic trauma is advised, with HBO2 considered if hearing does not improve within 7 days (63). Alternative immune-regulating pharmacotherapies, such as azathioprine and methotrexate, as well as immunotherapy with biopharmaceuticals such as etanercept, infliximab and adalimumab, have also shown promising results (29). Azathioprine and methotrexate inhibit cell proliferation through mechanisms involving purine synthesis suppression and disruption of DNA, RNA and protein synthesis via dihydrofolate reductase inhibition, respectively (64). On the other hand, etanercept, infliximab and adalimumab target TNF-α, effectively neutralizing its activity and reducing inflammation (65). Additionally, hindering proinflammatory cytokines and exploring autophagy also hold potential in reducing noise-induced threshold shifts (66).

Furthermore, another study demonstrated that mesenchymal stromal cell (MSC) therapy exhibits protective effects against NIHL (67). Through the RNA sequencing analysis of mice exposed to narrow band noise and treated with MSCs, the marked upregulation of genes related to immune modulation, hypoxia response and mitochondrial function was observed, alongside the downregulation of genes associated with synaptic remodeling and calcium homeostasis. These findings suggest that MSC treatment could offer a novel therapeutic approach for preventing or mitigating NIHL, contributing to innovative strategies for treating sound trauma-induced hearing loss and protecting hearing (67). Thus, future avenues for exploration include dietary micronutrient intake, gene therapies and regenerative medicine for NIHL prevention and treatment.

Clinical management strategies involve the use of hearing aids and/or hearing protection during noise exposure. In certain cases, cochlear implants are considered, provided that the cochlear nerve has not suffered severe damage.

## 9. Environmental and occupational considerations

### Role of environmental and occupational factors in NIHL

**Figure 2:**
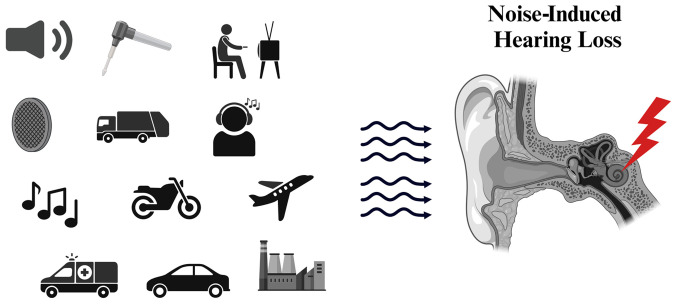
Contributing factors of NIHL. Noise originating from various sources causes auditory damage and leads to hearing loss. The type, intensity, duration and frequency of noise exposure all play crucial roles in the extent of the damage. Environmental noise from urban areas, recreational activities and community settings, combined with occupational noise from industries, construction, and service sectors, significantly contributes to NIHL. NIHL, noise-induced hearing loss. The figure was created using BioRender.com.

NIHL is markedly influenced by various environmental and occupational factors, each playing a critical role in both its onset and exacerbation of the condition (Fig. 2). Key considerations include noise levels and types, duration of exposure, the use of hearing protection and adherence to workplace regulations (68).

Prolonged exposure to high levels of noise, whether in the workplace or the surrounding environment, is a primary contributor to NIHL. Industries such as manufacturing, construction and aviation often expose workers to elevated levels of noise. The duration of exposure is crucial, as continuous or frequent exposure over extended periods of time increases the risk of developing NIHL. The nature of the noise, including its frequency and intensity, also plays a role, with high-frequency sounds and impulse noises being particularly damaging (69). In occupational settings, the use of personal protective equipment, such as earplugs or earmuffs can help mitigate the risk of NIHL, although consistent and proper use is essential. These devices function as barriers, reducing the intensity of sound reaching the ears and preventing damage to the auditory system (70). Adherence to safety regulations and guidelines related to noise exposure in the workplace is important, and employers should implement measures to reduce noise levels and provide training on the proper use of protective equipment.

Additionally, adopting situational awareness and consciously limiting exposure to loud environments, particularly during recreational activities, contributes to personal protection against NIHL. By integrating these measures into daily routines and adhering to recommended safety practices, individuals can proactively take steps to preserve their hearing health and prevent the adverse effects of excessive noise exposure. In the realm of personal audio device use, listening to music through headphones is a common practice that requires a nuanced approach to safeguard auditory health. Maintaining a moderate volume level, incorporating breaks to prevent ‘hearing fatigue’, and considering the use of noise-canceling or over-the-ear headphones are recommended strategies. These measures collectively foster an enjoyable music experience while prioritizing the preservation of auditory welfare. Furthermore, the regular monitoring of hearing health and prompt professional evaluation in response to any observed auditory irregularities contribute to a comprehensive preventative framework.

### The vulnerability of children to noise-induced damage impacts cognitive development and academic performance

NIHL in children poses a notable concern due to their developing auditory systems and frequent exposure to loud environments. In classrooms, they are surrounded by various noises, ranging from external disturbances such as road traffic and neighboring classroom activities to internal sources, such as electronic devices (i.e., printers), amidst the lively chatter and movements of children (71). Moreover, children often encounter unexpected sources of noise, including certain toys that exceed safe listening levels, further jeopardizing their hearing health (72). Their immature cochlear structures render them more susceptible to damage from prolonged exposure to high-volume sounds, potentially leading to irreversible hearing impairment and hindering their sound perception and processing abilities. Beyond auditory repercussions, NIHL in children can profoundly affect their overall growth and well-being. The susceptibility of children to noise-induced damage has notable implications for their intellectual growth and academic performance in school. Prolonged exposure to excessive noise, whether in the academic environment or through recreational activities, can disrupt the delicate process of cognitive development in children. Noise-induced stress and distractions may hinder concentration, memory retention and overall cognitive functioning, impacting their ability to excel academically (73).

Additionally, the potential for hearing damage poses a direct threat to language acquisition, communication skills and engagement in classroom activities. Recognizing and addressing the susceptibility of children to noise-induced damage is crucial for fostering a conducive learning environment and ensuring optimal cognitive growth during their formative years in school.

### Educational and awareness programs alongside preventive measures and regulations for reducing the risk of developing NIHL

Understanding the impact of environmental and occupational factors is essential for formulating effective strategies to prevent or minimize NIHL. Disseminating information about the risks of noise exposure and fostering awareness of preventive measures can markedly contribute to NIHL reduction, applicable not only in occupational settings, but also within the broader community. Another key aspect involves educating children, adolescents and young adults about hearing and the impacts of noise, aiming to influence their listening behaviors.

Piccino *et al* (74) recently investigated hearing health interventions to prevent NIHL among 41 school students aged 12-14 years. Their study employed pre- and post-study, and 4-month follow-up questionnaires to assess intervention impact. Initially, none of the students used earplugs at concerts or loud parties; however, post-intervention, usage increased to 19.5%, declining to 4.8% 4 months thereafter. Similarly, earplug use in noisy settings increased immediately after the intervention to 9.75%, sustaining >4 months. These results highlight effective knowledge dissemination and sustained improvements post-intervention. The active engagement of students has been crucial in raising awareness and initiating behavior changes, emphasizing the importance of ongoing educational efforts to address auditory behaviors among youth.

You *et al* (75) surveyed college students regarding their attitudes and behaviors related to hearing conservation and personal listening device (PLD) usage. Despite awareness of healthy hearing practices, numerous students use PLDs at high volumes in noisy environments without realizing the associated risks. However, there was a positive response towards receiving further information about hearing conservation. Students with a greater knowledge of hearing loss were more inclined to limit PLD usage, indicating the potential impact of education on promoting hearing conservation behaviors among young adults.

Preventive measures and regulations play a pivotal role in reducing the risk of developing NIHL. A comprehensive approach that combines legislative measures, public awareness campaigns and technological solutions is essential for effectively minimizing the risk of NIHL in various environments. Implementing and enforcing workplace noise exposure limits, along with providing and promoting the use of hearing protection devices, are crucial measures (76). Educational initiatives to raise awareness about the dangers of excessive noise, both in occupational and recreational settings, contribute to risk reduction. Moreover, incorporating engineering controls, such as noise barriers and soundproofing, further alleviates exposure. Regulatory frameworks that mandate noise control measures in industries and public spaces are instrumental in safeguarding individuals from NIHL.

Numerous European countries have implemented regulations to restrict the maximum volume level of PLDs, including smartphones, to 100 dBA since 2002. Furthermore, the US Occupational Safety and Health Administration has provided guidelines, cautioning against potential NIHL if music is listened to for >2 h at 100 dBA. In Korea, a prominent player in the information technology industry, smartphones have surged in popularity, offering an array of multimedia features. Notably, in 2014, the Korean government and phone manufacturers voluntarily agreed to limit the maximum volume level of mobile phones to 100 dBA during audio playback (77). Despite these regulatory measures, a number of users acknowledge surpassing recommended sound limits, particularly in noisy settings. The study conducted by Kim and Han disclosed that 60% of 1,480 respondents use their mobile phone programs for ≥1 h daily, with 10.8% extending usage to >3 h per day for audio listening (78).

## 10. Interdisciplinary collaboration, translational implications and future directions

The integration of expertise from diverse fields, including auditory physiology, genetics, pharmacology and clinical medicine, enables a comprehensive understanding of the complexities of NIHL. Fostering collaboration between researchers using animal models and those working on human applications facilitates the translation of insights gained from preclinical studies into effective clinical strategies. This interdisciplinary approach identifies commonalities and differences between species, enabling the development of targeted interventions and therapeutic modalities that are both scientifically rigorous and clinically relevant. The synergy of diverse perspectives and methodologies is paramount in advancing NIHL research, ultimately improving the translation of findings from bench to bedside.

The translational potential of recent research findings on NIHL holds significant promise for clinical applications, aiming to mitigate or prevent this prevalent and often preventable form of hearing loss. Investigating the roles of oxidative stress, inflammation and genetic susceptibility contributes to a deeper understanding of NIHL pathophysiology, potentially leading to the development of pharmaceutical interventions, gene therapies, or innovative hearing protection devices. Furthermore, the identification of biomarkers associated with NIHL may facilitate early detection and personalized treatment approaches. Overall, ongoing research on NIHL has the potential to bring about transformative clinical applications, addressing the complex challenges posed by this widespread condition.

## 11. Conclusions

The present review delved into the molecular and cellular mechanisms underlying NIHL and emphasized the importance of preventive measures and educational interventions. By providing insight into targeted treatments and shaping public health policies, it contributes to a nuanced understanding of NIHL and its management. This holistic approach offers valuable guidance for researchers, clinicians and policymakers in effectively addressing this prevalent auditory disorder.

## Figures and Tables

**Table I:** Comparison of model organisms in hearing research: Rodents, zebrafish and non-human primates.

Feature of the model organism	Rats	Mice	Zebrafish	Non-human primates
Cost-effectiveness, Availability	Widely available for large-scale experiments, cost-effective	Widely available for large-scale experiments, cost-effective	Cost-effective, rapid breeding and maintenance	Limited availability, higher cost, bridging rodent studies to human applications
Anatomical and physiological similarities of the auditory system to humans	Similar cochlear anatomy, physiology and pathology	Similar number of cochlear turns, but different cochlear duct length, higher hearing frequency range	Lack outer and middle ear, inner ear with hair cells and lateral line system with neuromasts, regenerative capabilities, absence of mammalian cochlea	Marked physiological and anatomical parallels
Testing capabilities	Electrophysiological hearing tests and behavioral assessments	Genetic and molecular insights into factors influencing NIHL	Real-time observation of inner ear development and function, hair cell regeneration	Advanced cognitive capacities, studies on complex auditory processing
Developmental considerations	Hearing matures postnatally	Genetically modifiable, allowing study of factors like sex, age and genetic background	Transparent embryos/larvae facilitate visualization of noise impacts on inner ear structures	Behavioral similarities aid in understanding auditory stimuli perception

NIHL, noise induced hearing loss.

## Data Availability

Not applicable.
